# A Newly Isolated *Streptomyces* sp. YYS-7 With a Broad-Spectrum Antifungal Activity Improves the Banana Plant Resistance to *Fusarium oxysporum* f. sp. *cubense* Tropical Race 4

**DOI:** 10.3389/fmicb.2020.01712

**Published:** 2020-08-12

**Authors:** Yuanyuan Wei, Yankun Zhao, Dengbo Zhou, Dengfeng Qi, Kai Li, Wen Tang, Yufeng Chen, Tao Jing, Xiaoping Zang, Jianghui Xie, Wei Wang

**Affiliations:** ^1^Key Laboratory of Biology and Genetic Resources of Tropical Crops, Ministry of Agriculture, Institute of Tropical Bioscience and Biotechnology, Chinese Academy of Tropical Agricultural Sciences, Haikou, China; ^2^College of Horticulture and Forestry Sciences, Huazhong Agricultural University, Wuhan, China; ^3^Haikou Experimental Station, Chinese Academy of Tropical Agricultural Sciences, Haikou, China

**Keywords:** *Streptomyces*, fusarium wilt of banana, antifungal activity, GC-MS, biocontrol

## Abstract

Fusarium wilt of banana caused by *Fusarium oxysporum* f. sp. *cubense* (Foc) is one of the most destructive diseases, severely limiting the development of banana industry. Especially, Foc tropical race 4 (Foc TR4) can infect and destroy almost all banana cultivars. Until now, there is still a lack of an effective method for controlling fusarium wilt. A biocontrol strategy using Actinobacteria is considered as a promising method for management of disease and pest. In this study, 229 Actinobacteria were isolated from rhizosphere soil samples of a primitive ecological mountain. An actinobacterium strain marked with YYS-7 exhibited a high antifungal activity against Foc TR4. Combining the physiological and biochemical characteristics as well as alignment of the 16S *rRNA* sequence, the strain YYS-7 was assigned to *Streptomyces* sp. The crude extracts of *Streptomyces* sp. YYS-7 obviously inhibited the mycelial growth of Foc TR4. The cell integrity and ultrastructure were seriously destroyed. In addition, *Streptomyces* sp. YYS-7 and crude extracts also showed a broad-spectrum antifungal activity against the selected seven phytopathogenic fungi. A gas chromatography-mass spectrometry (GC-MS) was used to predict the antifungal metabolites. A total of eleven different compounds were identified, including phenolic compounds, hydrocarbons, esters and acids. In the pot experiment, the crude extracts can significantly improve the banana plant’s resistance to Foc TR4. Hence, *Streptomyces* sp. YYS-7 will be a potential biocontrol agent for the biofertilizer exploitation and the discovery of new bioactive substances.

## Introduction

Plant diseases caused by various phytopathogens seriously result in the global crop yield reduction. Fungal pathogens are one of the major causative agents of plant diseases (Borrero et al., 2006). Currently, strategies of controlling plant fungal diseases are mainly application of some synthetic fungicides, including triazoles and acylalanines, etc. (Emmert and Handelsman, 1999). However, excessive use of agrochemicals not only causes environmental pollution and human health hazards, but also induces the resistance or reduces the susceptibility of pathogenic fungus. Safe and effective biocontrol methods have received more attention for management of various fungal diseases (Raza et al., 2016; Zapata-Sarmientoa et al., 2019).

Different microbial species such as bacteria, fungi and Actinobacteria have been successfully used for controlling plant pathogens (Supaphon et al., 2013; Raza et al., 2016; Soltanzadeh et al., 2016; Zapata-Sarmientoa et al., 2019). Especially, Actinobacteria are well-known prolific producers of bioactive secondary metabolites (Lam, 2006; Dewi et al., 2018). These secondary metabolites isolated from Actinobacteria account for 45% of natural products derived antimicrobial drugs (Valli et al., 2012). For example, some metabolites, known as antibacterial, antifungal, neuritogenic, anticancer, antialgal, antimalarial, and anti-inflammatory activities, have been widely used in agricultural, pharmaceutical and industrial fields (Leon et al., 2016). Moreover, Actinobacteria protect plants from a wide range of phytopathogenic fungi by the production of fungal cell wall degrading enzymes, antifungal antibiotics and plant growth promoters (Singh and Gaur, 2016; Singh et al., 2018; Aamir et al., 2020). As one of the most important genera of Actinobacteria, species of *Streptomyces* are the most abundant soil microorganisms under different ecological environments. Various secondary metabolites with novel structure and remarkable biological activities were identified and successfully developed into formulations to control fungal phytopathogens of various crops (Ayyadurai et al., 2006; Faheem et al., 2015; Goudjal et al., 2016; Lu et al., 2016; Duan et al., 2020). However, selection of these functional microbes is limited by their growth conditions and antifungal activities. Hence, it is important to isolate effective antagonistic *Streptomyces* against different phytopathogenic fungi.

Banana (*Musa* spp.) is one of the world’s most important fruits. Development of banana industry is seriously threatened by Fusarium wilt caused by the soil-borne fungus *Fusarium oxysporum* f. sp. *cubense* (Foc) (Wang et al., 2015). Especially, the Foc Tropical Race 4 (Foc TR4) is regarded as the most disastrous race and attacks almost all banana cultivars, thereby causing extensive destruction of banana orchards (Shen et al., 2015; Wang et al., 2017). Until now, there is no an effective physical or chemical strategy to prevent the spread of fungal disease. Considering the environmental pollution and human health, biocontrol is a promising method to control Fusarium wilt of banana. In the present study, an Actinobacterium with a high antifungal activity to Foc TR4 was isolated from soil samples of the primitive “Yingge” mountain. Based on analysis of morphological and biochemical characteristics as well as alignment of 16S *rRNA* sequence, the strain was designated as *Streptomyces* sp. YYS-7. Its crude extracts severely affected ultrastructures and integrity of Foc TR4 cells. Gas chromatography mass spectrometry (GC-MS) was used to predict antifungal compounds of crude extracts from *Streptomyces* sp. YYS-7. In addition, crude extracts also exhibited a broad-spectrum antifungal activity against the selected seven phytopathogenic fungi and improved the resistance of banana plant to Foc TR4. Hence, *Streptomyces* sp. YYS-7 will be a potential bioresource for controlling the different fungal diseases in the future application.

## Materials and Methods

### Soil Sample Collection

Rhizosphere soil samples of six plants (Supplementary Table S1) were collected from the primitive ecological nature reserve of “Yingge” mountain, Hainan Province, China. An approximately 10–20 cm soil layer was selected. The soil samples were immediately transported to the laboratory in sterile plastic bags and stored at −20°C for screening isolates.

### Phytopathogenic Fungi

Seven phytopathogenic fungi were selected to analyze the broad-spectrum antifungal activity, including *Fusarium oxysporum* f. sp. *cubense* Tropical Race 4 (Foc TR4, ATCC 76255), *Colletotrichum fragariae* (ATCC 58718), *Colletotrichum gloeosporioides* (ACCC 36351), *Fusarium oxysporum* f. sp. *cucumerinum* (ATCC 204378), *Colletotrichum acutatum* (ATCC 56815), *Colletotrichum musae* (ATCC 96726), *Curvularia fallax* (ATCC 34598), and *Fusarium graminearum* (DSM 21803). These phytopathogenic fungi were kindly provided by the Institute of Tropical Bioscience and Biotechnology, China Academy of Tropical Agricultural Sciences, Haikou, China.

### Isolation of Actinobacteria

Actinobacteria were isolated by a serial dilution method on different agar media, including glucose aspartic acid (GA) (Pridham and Lyons, 1961), humic acid-vitamin (HV) (Hayakawa and Nonomura, 1987), starch-casein (SCA) (Ruan et al., 1994), and Gause’s no. 1 (Williams et al., 1983), respectively. 50 mg L^–1^ of potassium dichromate and actidione were added to inhibit bacterial and fungal contamination. Soil samples were first dried at room temperature, then ground using a pestle and sieved with a 0.425 mm mesh. Three grams of dried soil were agitated vigorously in 27 mL of sterile water at 55°C for 20 min. Each soil suspension diluted from 10^–1^ to 10^–3^-fold was aseptically spread on different media and incubated at 28°C for 7–10 days. Some selected colonies were then inoculated onto the yeast extracte-malt extract agar (ISP2) for purification (Shirling and Gottlieb, 1966). The selected isolates were kept in slant agar media at −4°C, respectively. Each stock culture was preserved in 20% of glycerol (v/v) at −80°C.

### Analysis of Antifungal Activity

Isolates were screened for antifungal activity against Foc TR4 on the potato dextrose agar (PDA) plates using a conventional spot inoculation method (Sadeghian et al., 2016). Crude extracts from these isolates were tested using a disk diffusion method (Mohseni et al., 2013). Briefly, mycelium blocks (5 mm diameter) of the isolate were inoculated at four symmetrical points of the PDA plate. A phytopathogenic fungal disk (5 mm diameter) was placed in the center of the PDA plate. No isolate mycelium block was used as a control. After incubation at 28°C for 5–7 days, antifungal activity of the isolate was evaluated by measuring the diameters of inhibition zones (distance of the fungal mycelium to the isolate) and the percentage of fungal growth inhibition (GI) according to the following formula:

GI = [(D − D_1_)/D_1_] × 100%

Where D and D_1_ represented the diameters of fungal mycelium growth in the control and treated plates, respectively.

### Identification and Characteristics of Actinobacteria

Cultural characteristics of Actinobacteria were examined on various International Streptomyces Project (ISP) agar media, including PDA, Gause’s no. 1, tryptone-yeast extract (ISP1), ISP2, oatmeal (ISP3), inorganic salts (ISP4), glycerol asparagine (ISP5), peptone-yeast extract (ISP6), and tyrosine (ISP7) (Shirling and Gottlieb, 1966). The growth conditions, aerial mycelia, soluble pigment and colony profiles were assayed after incubation at 28°C for 5–7 days. The morphology of spore chains was observed by a scanning electron microscopy (SEM, model S-4800, Hitachi Limited, Japan). The growth characteristics of isolates were examined by changes of carbon and nitrogen utilization, pH range (4–10) and NaCl (1–11%, w/v) tolerance on the ISP2 medium. Some biochemical profiles including nitrate reduction, gelation liquefaction hydrolysis of cellulose, starch, tween 20, tween 40, tween 80, H_2_S production, and urease activity were also measured according to the previous description of Li et al. (2016). Resistance evaluations of each isolate to 30 standard antibiotics were tested by a disk diffusion method (Bakht et al., 2014).

### DNA Extraction and PCR Amplification

The isolate was cultured in the ISP2 liquid medium at 28°C for 4 days. Total genomic DNA was extracted using a Rapid Bacterial Genomic DNA Isolation Kit (Bioteke Corporation, Beijing, China) according to the standard manufacturer protocol. A 16S *rRNA* gene of the isolate was amplified using a pair of universal primers (27F: 5′-AGAGTTTGATCCTGGCTCAG-3′, 1492R: 5′-GGTTACCTTGTTACGACTT-3′) (Wang et al., 2015). The reaction system contained 2 μL of genomic DNA, 25 μL of 2 × Taq Master Mix, 2 μL of 27F primer, 2 μL of 1492R primer and 19 μL of ddH_2_O. The PCR reaction was performed in a Veriti thermal cycler (Applied Biosystems, Carlsbad, CA, United States) with an initial denaturation step at 94°C for 5 min, followed by 32 cycles of denaturation at 94°C for 1 min, annealing at 55°C for 1 min and extension at 72°C for 2 min with a final extension step at 72°C for 10 min. Finally, the PCR product was detected by 1% (w/v) of agarose gel electrophoresis and was sequenced by the Shanghai Sangon Biotech Co., Ltd. (Shanghai, China).

### Construction of Phylogenetic Tree

The sequenced 16S r*RNA* of the isolate was aligned against the GenBank database^1^ and the EzBioCloud server^2^ to obtain the homology sequences. The multiple sequence alignment was performed by the CLUSTAL W program of BioEdit 7.0.5.3 (Saitou and Nei, 1987). A phylogenetic tree was constructed using the neighbor-joining method of MEGA version 7.0 (Kumar et al., 2016). The support of each clade was determined by a bootstrap analysis performed with 1000 replications. A distance matrix was generated using a Kimura’s two-parameter model. All positions containing gaps and missing data were eliminated from the dataset (complete deletion option).

### Preparation of Crude Extracts of Actinobacteria

Crude extracts were obtained from the isolate according to the previous description of Sajid et al. (2009). Briefly, the isolate was incubated in a sterilized soybean liquid medium at 150 rpm and 28°C for 7 days. After filtration, the evaporated fermentation broth was subjected to a silica gel chromatography column (100–200 μm particle size, 5.5 × 30 cm). The elution was performed using a gradient of methanol (50, 60, 70, and 100%) with a flow rate of 2 mL min^–1^. Crude extracts isolated by four methanol gradients were dissolved in 10% of dimethyl sulfoxide (DMSO), respectively, obtaining 20 mg mL^–1^ of stock solution. 100 μg mL^–1^ of crude extracts were added to the autoclaved PDA medium. 10% of DMSO was used as a control. Each experiment was carried out in triplicate. After incubation at 28°C for 5–7 days, the antifungal activities and the average inhibition rates were calculated.

### Toxicity Assays

Based on *in vitro* antifungal activity assay, crude extracts of the isolate were used to determine the toxicity against Foc TR4. The stock solution was diluted to 200, 100, 50, 25, 12.5, 6.25, 3.125, 1.5625, and 0.78 μg/mL, respectively. Four wells (6 mm in diameter) with 26 mm from the center were punched by a sterile cork borer at the PDA solid medium (90 mm diameter). 50 μL of different dilutions were added to each well. Equal amount of DMSO (10%, v/v) was used as a control. Subsequently, a fungal disk (5 mm in diameter) was placed in the center of each petri dish aseptically. All plates were incubated at 28°C until the mycelium growing to the edge of plate in the control group. Diameters of these inhibition zones were measured. A linear regression, namely a toxicity regression equation, was established by a least square method (Yun et al., 2018). EC_50_ and EC_95_ values were calculated from the toxicity regression equation. All experiments were performed in three biological replicates.

### Effect of the Isolate on Growth of Fungal Mycelium

The seven fungal phytopathogens were selected to assess antifungal activity of the isolate by using a conventional spot inoculation method (Shomura et al., 1979). Each mycelium block (5 mm diameter) of the isolate was inoculated at four symmetrical points of the PAD plate. A phytopathogenic fungal disk (5 mm diameter) was placed in the center of plate. After incubation at 28°C for 5–7 days, antifungal activity was evaluated by measuring the inhibition zone (distance of the fungal mycelium to the isolate) around the mycelial block. The percentage of fungal growth inhibition was calculated according to the following formula:

Growth inhibition percentage = [(D − D_1_)/D] × 100

Where D and D_1_ represented the growth diameters of fungal mycelia in the control and treated plates, respectively.

### Effect of Crude Extracts on Mycelial Growth of Phytopathogens

The inhibition activity of crude extracts on mycelial growth was assessed by an agar well-diffusion method (Atta, 2015). 10 mg of crude extracts were dissolved in 1 mL of DMSO (10%, v/v) as a stock solution, followed by sterilization using a 0.22 μm membrane filter. Four wells (6 mm diameter) were punched by a sterile cork borer at the PDA agar medium (90 mm diameter). Then, 50 μL of crude extracts were added to each well. Equal amount of DMSO (10%, v/v) was used as a control. A fungal disk (5 mm diameter) was placed on the center of each petri dish aseptically. All plates were incubated at 28°C until the control mycelium growing to the edge of plate. The inhibition zones were measured by a cross method (Qi et al., 2019). The percentages of fungal growth inhibition were calculated separately according to the following formula:

Growth inhibition percentage = [(D − D1)/D] × 100

Where D and D1 were the diameters of phytopathogenic fungal mycelia in the control and treated plates, respectively.

### Determination of Minimum Inhibitory Concentration (MIC) and Minimum Fungicidal Concentration (MFC) of the Isolate

Minimum Inhibitory Concentrations of crude extracts from the isolate against eight phytopathogenic fungi were measured using a 96-well plate (Nunc MicroWell, Roskilde, Denmark) (Wang et al., 2013; Song et al., 2016). Two-fold serial dilutions of crude extracts were prepared for the MIC tests (50.00–0.04 μg mL^–1^). Each well contained 80 μL of mycological media of the Roswell Park Memorial Institute (RPMI), 100 μL of fungal suspension with 1.0 × 10^5^ CFU mL^–1^ and 20 μL of crude extract solution. Two standard antibiotics of cycloheximide and nystatin were served as positive controls. Equal volume of DMSO (10%, v/v) was used as a negative control. MICs were measured according to the previous description of De Toledo et al. (2016). Each sample in the 96-well plate was inoculated on the PDA medium at 28°C for 4 days. The concentration completely inhibiting the fungal growth was defined as the MFC value. Each experiment was performed with three biological replicates.

### GC-MS Analysis of Crude Extracts

Component analysis of crude extracts was carried out on a Trace-DSQ-GC-MS system (Thermo Fisher Scientific, Waltham, MA, United States). The GC-MS analysis was performed with a Trace GC coupled to a Trace DSQII quadrupole mass spectrometer (Thermo-Fisher Scientific, Waltham, MA, United States) and equipped with a DB-5MS capillary column (30 m length, 0.25 μm thickness and 0.25 mm internal diameter, J & W Scientific, United States). The sample was dissolved into the spectroscopy-grade methanol, filtering through a 0.2 μm filter. 1 μL of sample was injected into the heated injector (250°C) using helium as a carrier gas at flow rate of 1 mL min^–1^. The oven procedure started at 60°C for 1 min, following a temperature ramp of 5°C min^–1^ to 100°C with a hold for 5 min, and then again raised to 250°C with a second ramp of 10°C min^–1^ with a hold for 35 min, furthermore, again increased to 280°C with a three ramp of 8°C min^–1^ with a final hold for 25 min. The mass spectrometer was operated in the electron ionization mode at 70 eV with a continuous scan from 50 to 650 m/z. Compounds were identified by matching the mass spectra with the MS spectral database (NIST spectra library program version 2.0; Thermo-Fisher Scientific, Waltham, MA, United States). A triplicate analysis was performed on each sample.

### Effect of Crude Extracts on Spore Germination of Foc TR4

The Foc TR4 was cultured on the PDA medium at 28°C for 5–7 days. The fungal spores were obtained by rubbing and washing the surface of each petri dish with a sterile L-shaped spreader. The spore suspension was filtered through a sterile muslin to remove mycelia. The spore concentration was determined using a Haemocytometer (Neubauer, Superior Ltd., Marienfeld, Germany) and adjusted to a final concentration of 10^5^ CFU mL^–1^. The crude extracts diluted to 6.25, 12.5, 25, 50, 100, 200, 400, and 600 μg mL^–1^ were mixed with the spore suspension at a ratio of 1:1 (v/v), respectively. The mixture in a cavity glass slide was incubated in a moist chamber at 28°C for 12 h. A mixture of DMSO and spore suspension was used as a control. One hundred spores in each slide were observed by an optical microscope (Axio Scope A1, Carl ZEISS, Germany). The percentage of spore germination was calculated using the formula: PSG = (A−B)/A × 100%, where A and B represented the spore germination rates in the control and treatment groups, respectively.

### Detection of Morphology and Ultrastructures of Foc TR4 Cells

The morphology of Foc TR4 cells was detected after treatment with crude extracts of the isolate using a scanning electron microscopy (SEM, model S-4800, Hitachi Limited, Japan). One milliliter of Foc TR4 (1.0 × 10^5^ spores mL^–1^) was inoculated with 25 μg mL^–1^ of crude extracts for 24 h. The spores were fixed with 2.5% (v/v) of glutaraldehyde in a phosphate-buffered saline solution (0.1 mol L^–1^, pH 7.0, PBS) for 2 h and dehydrated using a series of increasing concentrations of ethyl alcohol (30–100%, v/v) for 10 min. Samples coated with a film of gold-palladium alloy under vacuum were detected by SEM (Ruiz et al., 2016). To evaluate effects of crude extracts on cell ultrastructures, the Foc TR4 sample was fixed with OsO_4_ (1%, w/v) in 0.1 mol L^–1^ of PBS for 1 h at room temperature, then dehydrated by a gradient solution of methanol (50 to 100%) for 10 min (Lou et al., 2011). The samples were embedded in a spurr resin and cut with an Ultracut Ultramicrotom (EM UC6, Leica, Germany). These sections were stained with the saturated uranyl acetate and the lead citrate and observed by a Transmission Electron Microscope (TEM, JEM-1400 Flash, Hitachi Limited, Tokyo, Japan).

### Banana Plantlets Inoculated With Foc TR4

A pot experiment was carried out for detecting the fermentation broth role of the isolate on improving banana plantlets’ resistance to Foc TR4. The isolate was cultured in a sterilized soybean liquid medium at 150 rpm min^–1^, 28°C for 5–7 days. The fungal spores of Foc TR4 (10^6^ CFU mL^–1^) were inoculated to the roots of banana plantlets as our previously described method (Wang et al., 2012). And then, the fermentation broth of the isolate (10^6^ CFU g^–1^ soil) was also added to each plant. The medium and sterile water treatments were used as controls. The banana plantlets were transferred to a greenhouse at 28 ± 2°C for 5 weeks. After 2, 7, 14, 21, and 28 days, the chlorophyll contents and height of banana plantlets were measured (Yoon et al., 2017). Leaves were photographed and chlorotic areas (expressed as percentage for a whole leaf) were quantified using the Image J software^3^. Data were acquired from three independent experiments and 30 leaves were selected for each experiment.

### Statistical Analysis

The EC_50_ and EC_95_ values were calculated from the linear regression analysis. The pot experiment was carried out using a completely randomized design. Statistical analysis was performed with the SPSS Version 13.0 software (SPSS Inc., Chicago, IL, United States). All data were expressed as means ± the standard error (SE) from three biological replicates of each experiment. Significant difference between means was determined by the Tukey’s *post hoc* comparison test at *p* < 0.05.

## Results

### Isolation of Actinobacteria From the Rhizosphere Soil

**FIGURE 1:**
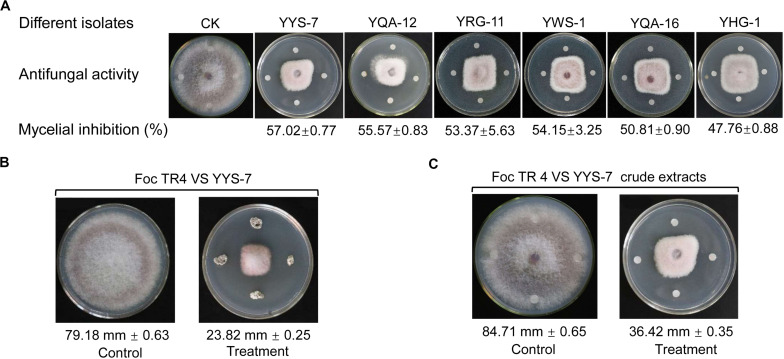
Effects of different isolates on the mycelial growth of Foc TR4. **(A)** Antifungal activities of six isolates against Foc TR4. **(B)** The strain YYS-7 obviously inhibited the mycelial growth of Foc TR4. **(C)** Crude extracts of the strain YYS-7 significantly inhibited the mycelial growth of Foc TR4.

A total of 229 actinomycete isolates with different colony morphology were successfully isolated from the rhizosphere soil samples of six plants in the primitive ecological nature reserve (Supplementary Table S1). All isolates were screened in the light of their antagonistic activities against Foc TR4. A total of 20 isolates exhibited antifungal activity. Out of those, six isolates had a great potential for antifungal production with the larger inhibition zones (Figure 1A). Especially, an isolate numbered with YYS-7 had the strongest antifungal activity. Compared with the growth diameter (79.18 mm ± 0.63) of Foc TR4 in the control plate, the inhibition zone after treatment with the strain YYS-7 was reduced to 23.82 mm ± 0.25 (Figure 1B). Similarly, we also found that crude extracts of the strain effectively inhibited the growth of Foc TR4 and the inhibition zone was 36.42 ± 0.35 mm (Figure 1C). The inhibition percentages of mycelial growth were 69.91 and 57.02%, respectively. Therefore, the strain YYS-7 was selected and analyzed in the following experiment.

### Identification and Morphological Characteristics of the Strain YYS-7

After the strain YYS-7 was cultured on seven different media for 7 days at 28°C, we evaluated its growth conditions and morphological characteristics of aerial hyphae, vegetative mycelia, soluble pigment and colony (Supplementary Table S2). The strain YYS-7 can grow well on eight media (Table 1). No diffusible pigments were produced. The rectiflexibile spore chains with a rugose surface were observed using SEM (Supplementary Figure S1A). Aerial mycelia of the strain YYS-7 were largely generated on most of media. A number of vegetative mycelia were also detected. The characteristics of unfragmented substrate and aerial mycelia with long spore-chains suggested that the strain YYS-7 belonged to *Streptomyces* sp.

**TABLE 1:** Cultural characteristics of the strain YYS-7.

Culture medium	Aerial hyphae	Vegetative mycelium	Soluble pigment	Colony characteristics	Growth conditions
ISP2	White	Lemon Chiffon	None	Rigid and Plicate	+++
ISP3	Gray	Grainsboro	None	Dusty and Dry	+
ISP4	Cream	Corn silk	None	Pyknotic and Plicate	+++
ISP5	Cream	Lemon Chiffon	None	Dusty and Dry	+
ISP6	Oldlace	Peachpuff	None	Rigid and Plicate	+++
ISP7	Corn silk	Beige	None	Dry, Rigid and Plicate	+
Gause’s no. 1 medium	White	Lemon Chiffon	None	Rigid and Plicate	+++
PDA	Gray	Wheat	Pink	Dusty and Dry	+++

*+++, Good growth; ++, Moderate growth; +, Poor growth.*

A 1403 bp sequence of 16S *rRNA* of the strain YYS-7 was amplified and submitted to the GenBank database of NCBI with an accession number of MN397826. A phylogenetic tree was constructed using the Neighbor-Joining method with 1000 bootstrap replicates in MEGA (Version 7.0). The 16S *rRNA* sequence of the strain YYS-7 showed a high similarity with *Streptomyces albospinus* NBRC (AB184527) (Supplementary Figure S1B). Combining the morphological characteristics with model organisms of the genus, the strain YYS-7 was assigned to *Streptomyces* sp*..*

### Physiological and Biochemical Characteristics

To obtain antimicrobial compounds with high antifungal activities, the growth condition of *Streptomyces* sp. YYS-7 was optimized. The strain was able to grow at 13% of NaCl (moderate growth), but was unable to grow below pH 5.0. By contrast, optimal growth condition of the strain was at 30°C, pH 7.0 and 3% (w/v) of NaCl. Additionally, the physiological and biochemical characteristics of *Streptomyces* sp. YYS-7 were detected (Table 2). *Streptomyces* sp. YYS-7 could produce reductive nitrate, urease and gelatin as well as degrade tween 20. No H_2_S production and activity of cellulolytic enzyme were detected. It could utilize D-fructose, D-xylose, rhamnose, arabinose, melezitose, galactose, α- lactose, sucrose, D-mannose, sorbitol, inositol, sorbitol, D-mannitol, maltose, starch, and melibiose as a sole carbon source (Supplementary Table S3). Interestingly, *Streptomyces* sp. YYS-7 can grow in all tested nitrogen sources, including histidine, methionine, serine, glycine, arginine, valine, tyrosine, asparagine, L-phenylalanate and anhydrous creatine. In the evaluation of antibiotic sensitivity, the strain YYS-7 showed a wide resistance to clindamycin, chloramphenicol, norfloxacin, neomycin, doxycycline, gentamicin, ceftazidime, cefradine, cefazolin, midecamycin, oxacillin, and piperacillin, respectively (Supplementary Table S4). A sensitivity response was detected to furazolidone, compound sulfamethoxazole, polymyxin B, vancomycin, ciprofloxacin, ofloxacin, penicillin, erythromycin, minocycline, tetracycline, kanamycin, amikacin, cefoperazone, ceftriaxone, cefuroxime, cephalexin, carbenicillin, and ampicillin, respectively.

**TABLE 2:** Physiological and biochemical characteristics of the strain YYS-7.

Characteristic	Result
**Physiological**	
pH range for growth	4–10
Optimum pH for growth	7
NaCl tolerance	Up to 5
**Biochemical**	
Starch hydrolysis	+
Gelatin hydrolysis	+
Cellulose hydrolysis	-
Nitrate reduction	+
H_2_S production	+
Urease test	+
Twain 20	+
Twain 40	-
Twain 80	-
M-R test	-
V-P test	-

### Effect of Crude Extracts on Antifungal Activities of Foc TR4

**FIGURE 2:**
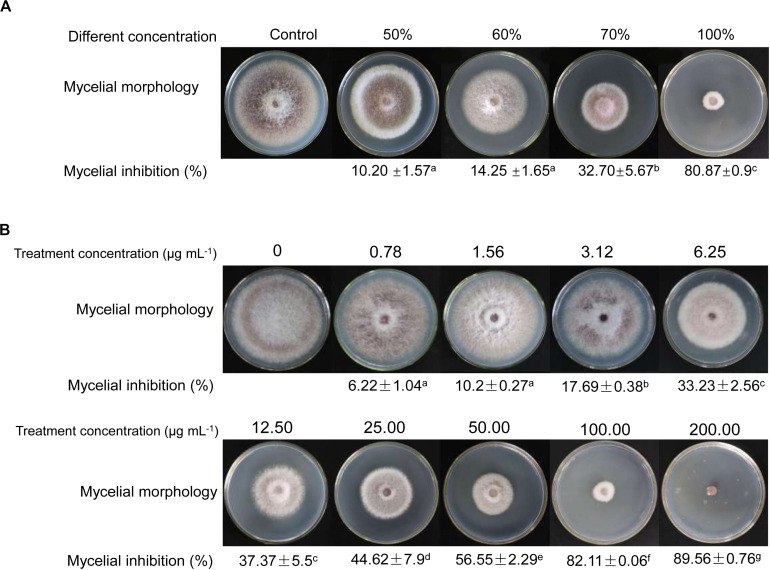
Antifungal activities of crude extracts on the mycelial growth of Foc TR4. **(A)** Antifungal activities of crude extracts isolated by different gradient methanol solvents. **(B)** Effects of different concentrations of crude extracts on mycelial growth of Foc TR4.

Compared with antifungal activities of crude extracts of *Streptomyces* sp. YYS-7 isolated with different concentration methanol, crude extracts isolated by 100% of methanol exhibited the strongest antifungal activity against Foc TR4 (Figure 2A). The inhibition rate of mycelial growth was 80.87%. The lowest antifungal activity with 10.21% was detected in crude extracts isolated by 50% of methanol. No obvious difference was found in antifungal activities of crude extracts isolated with 50% and 60% of methanol. Based on the results of toxicity assay, a significant linear correlation was obtained between the inhibition rate and log[concentration] value (*R*^2^ = 0.9857, *p* < 0.01) (Figure 2B). EC_50_ and EC_95_ values of crude extracts against Foc TR4 were 21.07 and 619.59 μg mL^–1^, respectively.

### Effect of Crude Extracts on Spore Germination and Morphology of Foc TR4

**FIGURE 3:**
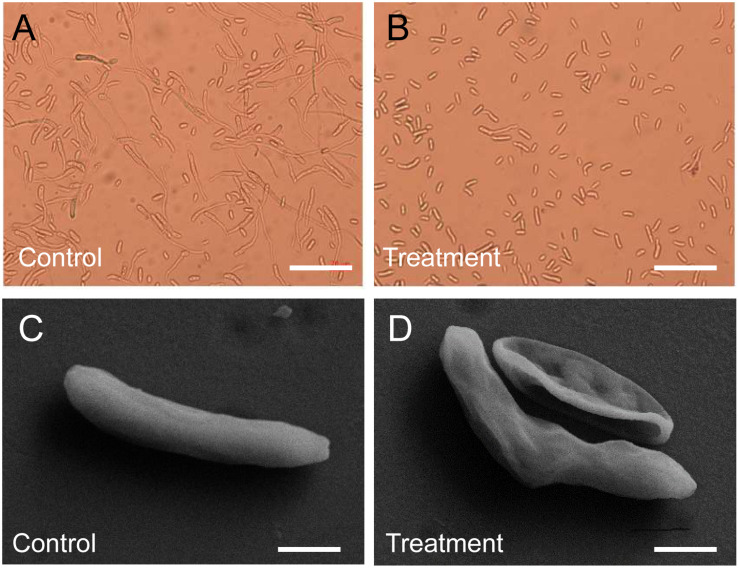
Effect of crude extracts on spore germination and morphological characteristics of the strain YYS-7. **(A)** Spore germination of Foc TR4 treated with 10% of DMSO for 24 h. **(B)** Crude extracts (600 mg mL^– 1^) effectively inhibited spore germination of Foc TR4. **(C)** Morphological characteristics of Foc TR4 spores treated with 10% of DMSO for 24 h. **(D)** Crude extracts (25 mg mL^– 1^) caused the morphological deformation of Foc TR4 spores.

Different concentration crude extracts of *Streptomyces* sp. YYS-7 were used to analyze the effects on spore germination and morphology of Foc TR4. By contrast, 50 μg mL^–1^ of crude extracts inhibited more than 50% of spore germination (Supplementary Table S5). With the concentration increase of crude extracts, the inhibition rate of spore germination was gradually enhanced. Especially, up to 91.50% of spore germination were inhibited after treatment with 600 μg mL^–1^ of crude extracts. When treated with 25 μg mL^–1^ of crude extracts for 24 h, the germ tube elongation was significantly inhibited (Figures 3A,B). Foc TR4 conidia showed wrinkled and deformity morphological profiles (Figures 3C,D). Intact shape and smooth surface were observed in the control conidia treated with 10% of DMSO.

### Effect of Crude Extracts on Ultrastructure of Foc TR4 Cells

To further evaluate antifungal activity of *Streptomyces* sp.YYS-7, ultrastructure changes of Foc TR4 were detected by TEM. In the control group, the cytoderm and membrane were intact and well defined (Supplementary Figure S2A). The organelles such as mitochondria, cell nucleus and vacuole were regularly arranged and the electron density in the cytoplasm was homogeneous. Foc TR4 cells treated with crude extracts of *Streptomyces* sp. YYS-7 exhibited some organelles in the cytoplasm were disintegrated and cell membrane and cytoderm were dissolved (Supplementary Figures S2B–F). In contrast, the treated Foc TR4 cells exhibited abnormal morphological characteristics, such as disappearing organelles and heterogeneity electron density in the cytoplasm.

### Analysis of a Broad-Spectrum Antifungal Activity of *Streptomyces* sp. YYS-7

**FIGURE 4:**
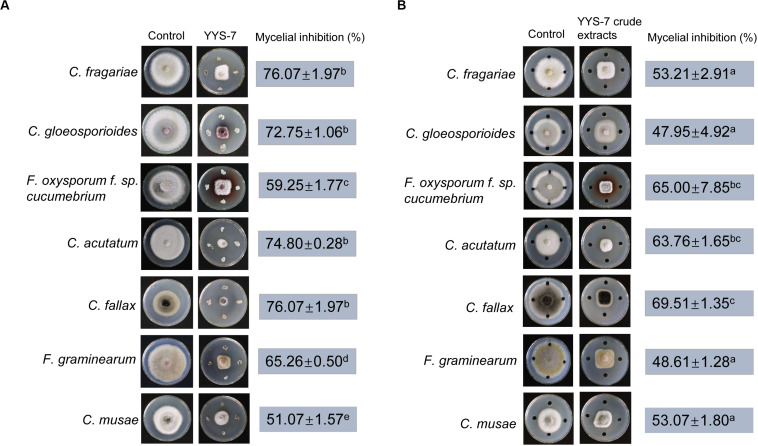
A broad-spectrum antifungal activity of *Streptomyces* sp. YYS-7 against phytopathogenic fungi. **(A)** Antifungal activities of the strain YY-7 against the selected phytopathogenic fungi. **(B)** Antifungal activities of crude extracts against the selected phytopathogenic fungi.

To assess whether *Streptomyces* sp. YYS-7 has a broad-spectrum antifungal activity, the seven phytopathogenic fungi were selected. The maximum inhibition percentage of mycelial growth was *C. fallax* (76.07 ± 1.97), while the lowest activity was detected against *C. musae* (51.07 ± 1.57). In addition, *Streptomyces* sp. YYS-7 also significantly inhibit mycelial growth of other phytopathogenic fungi (Figure 4A). The antifungal activities were showed as follows: *C. fragariae* (64.50 ± 0.7), *C. gloeosporioides* (72.75 ± 1.06), *F. oxysporum cucumerinum* (59.25 ± 1.77), *C. acutatum* (74.80 ± 0.28) and *F. graminearum* (65.26 ± 0.5).

Moreover, crude extracts of *Streptomyces* sp. YYS-7 were also used to detect the antifungal activities against the selected seven phytopathogenic fungi (Figure 4B). The maximum inhibition percentage of mycelial growth was found in *C. fallax* (69.51 ± 1.35). The lowest antifungal activity was exhibited against *C. gloeosporioides* (47.95 ± 4.92). The high inhibition percentages of mycelial growth were also detected in other selected fungi, such as *C. fragariae* (53.21 ± 2.91), *F. oxysporum cucumerinum* (65 ± 7.85), *C. acutatum* (63.76 ± 1.65), *F. graminearum* (48.61 ± 1.28), and *C. musae* (53.07 ± 1.80). The above results suggested that *Streptomyces* sp. YYS-7 has a broad-spectrum antifungal activity.

### MICs and MFCs of *Streptomyces* sp. YYS-7 Against Different Phytopathogenic Fungi

The MIC values of crude extracts against the eight phytopathogenic fungi were determined by a 96-well microtiter assay, ranging from 0.049 to 50 μg mL^–1^. The lowest MIC with 0.781 μg mL^–1^ was detected against *C. fallax*, suggesting that crude extracts had a high antifungal activity against this fungus. In addition, crude extracts also exhibited a strong antifungal activity against *C. fragariae*, *C. gloeosporioides*, *F. oxysporum cucumerinum*, *F. graminearum*, Foc TR4 and *C. musa*e with 1.5625, 3.125, 12.5, 3.125, 6.25, and 12.5 μg mL^–1^ of the MIC values, respectively. The highest MIC with 50 μg mL^–1^ was observed against *C. acutatum* (ATCC 56815) (Table 3). In the control group, the treatment of DMSO (10%, v/v) had no an obvious inhibition role for the selected seven phytopathogenic fungi.

**TABLE 3:** MIC of crude extracts of *Streptomyces* sp. YYS-7 against eight phytopathogenic fungi.

Pathogenic fungi	MIC of YYS-7 (μg mL^–1^)	MIC of Ny (μg mL^–1^)	MIC of Cy (μg mL^–1^)
*C. fragariae* (ATCC 58718)	>1.5625	>6.25	>0.098
*C. gloeosporioides* (ACCC 36351)	>12.5	>12.5	>3.125
*F. oxysporum* f. sp. *Cucumerinum* (ATCC 204378)	>3.125	>12.5	>3.125
*C. acutatum Simmonds* (ATCC 56815)	>50	>50	>50
*C. fallax* (ATCC 34598)	>0.781	>3.125	>0.049
*F. graminearum Schwabe* (DSM 21803)	>3.125	>0.049	>6.25
Foc TR4 (ATCC 76255)	>6.25	>6.25	>0.049
*Colletotrichum musae* (ATCC 96726)	>12.5	>3.125	>0.049

*Cy, cycloheximide (antifungal agent); Ny, nystatin (antifungal agent).*

We also determined the MFC values of *Streptomyces* sp. YYS-7 against eight phytopathogenic fungi. The MFC value was defined as the minimum concentration completely inhibiting the growth of fungus on medium. The MFC values of crude extracts ranged from 0.049 to 50 μg mL^–1^. The lowest MFC (6.25 μg mL^–1^) was detected against *C. fragariae* (ATCC 58718). Similarly, crude extracts also exhibit strong fungicidal activities against *F. oxysporum cucumerinum*, *C. fallax*, Foc TR4 and *C. musae*, respectively (Table 4). The highest MFC with 50 μg mL^–1^ was detected against *C. gloeosporioides*, *C. acutatum* and *F. graminearum*.

**TABLE 4:** MFC of crude extracts of *Streptomyces* sp. YYS-7 against eight phytopathogenic fungi.

Pathogenic fungi	MFC of YYS-7 (μg mL^–1^)	MFC of Ny (μg mL^–1^)	MFC of Cy (μg mL^–1^)
*C. fragariae* (ATCC 58718)	>6.25	>12.5	>25
*C. gloeosporioides* (ACCC 36351)	>50	>25	>50
*F. oxysporum* f. sp. *Cucumerinum* (ATCC 204378)	>25	>12.5	>25
*C. acutatum* (ATCC 56815)	>50	>3.125	>0.78
*C. fallax* (ATCC 34598)	>25	>3.125	>6.25
*F. graminearum* (DSM 21803)	>50	>12.5	>50
Foc TR4 (ATCC 76255)	>25	>25	>50
*C. musae* (ATCC 96726)	>25	>12.5	>0.049

*Cy, cycloheximide (antifungal agent); Ny, nystatin (antifungal agent).*

### Analysis of Crude Extracts by GC-MS

The GC-MS analysis was performed to predict the possible antifungal compounds of *Streptomyces* sp. YYS-7. Based on the information of retention time, molecular mass, molecular formula and structures, a total of eleven chemical compounds were identified by matching the mass spectra with the MS spectral database (Supplementary Table S6). These predicted compounds contained hexadecanoic acid, methyl ester, 2,4-di-tert-butylphenol, methyl stearate, tetradecanoic acid, dibutyl phthalate, pentadecanoic acid, n-hexadecanoic acid, cetene, phenol, 2,2′-methylenebis[6-(1,1-dimethylethyl)-4- methyl-, cyclohexanebutanoic acid and hexadecanoic acid (Supplementary Figure S3). The peak area represented a quantitative proportion of the predicted compound to the total of crude extracts.

### Evaluation of *Streptomyces* sp. YYS-7 on Improving the Plant Resistance to Foc TR4

**FIGURE 5:**
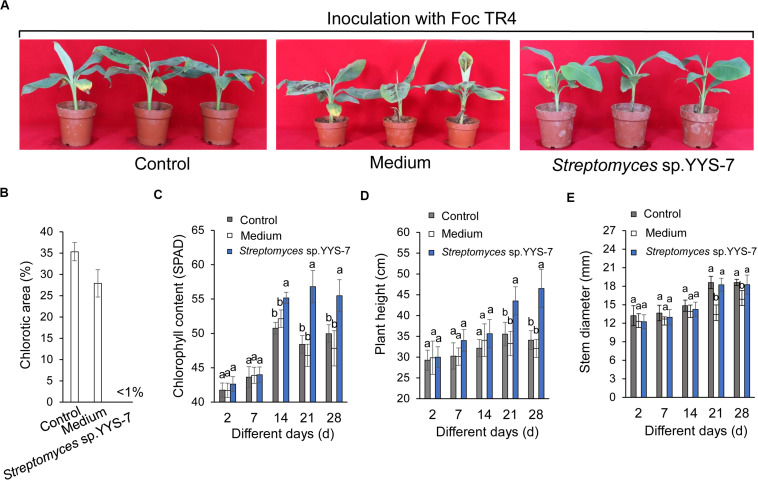
Crude extracts of *Streptomyces* sp. YYS-7 improving the banana plant’s resistance to Foc TR4. **(A)** Leaf chlorosis of banana treated with *Streptomyces* sp. YYS-7 28 days post Foc TR4 inoculation. Data indicate means ± SE from 21 leaves. **(B)** Quantification of chlorotic areas (expressed as percentages of total leaf areas) 28 days post inoculation. **(C)** Measure of chlorophyll contents of banana plantlets treated with *Streptomyces* sp. YYS-7 different days post Foc TR4 inoculation. **(D)** Measure of plant heights treated with *Streptomyces* sp. YYS-7 different days post Foc TR4 inoculation. **(E)** Measure of stem diameters treated with *Streptomyces* sp. YYS-7 different days post Foc TR4 inoculation. Columns represent means ± SE from 21 leaves from three independent repeats.

A pot experiment was performed to test whether the fermentation broth of *Streptomyces* sp. YYS-7 can enhance the plant resistance to Foc TR4. After co-inoculation with the fermentation broth and Foc TR4, the chlorotic symptom of banana leaves was detected. High chlorotic symptom of banana leaves in both water (25.32%) and sterilized soybean liquid medium treatment groups (24.89%) was observed after 28 days (Figure 5A). No chlorotic symptom was found in the leaves treated with *Streptomyces* sp. YYS-7 (Figure 5B). Therefore, the fermentation broth obviously improved the plant resistance to Foc TR4. Compared with the control and medium treatment groups, the fermentation broth effectively improved the chlorophyll contents, plant heights and stem diameters of banana plantlets after 14 days (Figures 5C–E). No significant differences were observed among different treatments before 14 days.

## Discussion

Plant fungal diseases caused serious losses and rot of fruits and vegetables (Duan et al., 2013). Fusarium wilt limited badly the continuous development of banana industry. Microbial antagonist was considered as the promising biocontrol strategy due to the characteristics of high efficiency, broad spectrum and environmental friendliness (Guo et al., 2013). The accumulated evidences indicated that Actinobacteria are the most potential producers of small diversity molecules (Shivlata and Satyanarayana, 2015). However, only few of Actinobacteria was identified and employed due to their differences of growth conditions and antagonistic activities. Therefore, isolation and screening of highly efficient antagonistic Actinobacteria are key to develop some biocontrol agents. In our study, the strain YYS-7 was isolated by analyzing the antifungal activity to Foc TR4. Based on alignment of 16S *rRNA* sequence and analysis of morphological and biochemical characteristics, the strain had the highest similarity with *S. albospinus* NBRC (AB184527), and was defined as *Streptomyces* sp. YYS-7.

*Streptomyces* sp. YYS-7 exhibited a broad-spectrum antagonistic activity against different phytopathogen fungi. Our previous work also showed that *Streptomyces* sp. SCA3-4 from the rhizosphere soil of *Opuntia stricta* had a strong antagonistic ability against Foc TR4 and other pathogen fungi (Chen et al., 2013; Qi et al., 2019). It suggested that the bioactive metabolites with antimicrobial activities can be produced by *Streptomyces* sp. For example, *S. padanus* PMS-702 produced a polyene macrolide antibiotic fungichromin, displaying high antagonistic activities against different phytopathogenic fungi (Fan et al., 2019). An antagonistic compound of 6-prenylindole isolated from *Streptomyces* sp.TP-A0595 inhibited the hyphae formation of *Alternaria brassicicola* (Sasaki et al., 2002). *S. griseus* H7602 owned a strong inhibitory activity against *Phytophthora capsici* (Nguyen et al., 2012).

In our study, we detected the morphological characteristics of Foc TR4 after treatment with crude extracts of *Streptomyces* sp. YYS-7 by SEM and TEM. Extensive degradation of cell wall and membrane was found and some organelles disappeared, resulting in the inhibition of mycelial growth. Similar results were also observed in cells of *Phytophthora capsici* and *P. cactorum* after exposure to fungicide (Brewster et al., 1997; Xu et al., 2007). The destroyed cell integrity and organelles may be involved in the response of fungal defenses (Yang et al., 2010). However, the specific antifungal mechanism still needs be further investigated.

Compared with the previous results, crude products of *S. lavendulae* strain SCA5 isolated with ethyl acetate demonstrated an antifungal activity with a MIC value of 31.25 μg/mL (Kumar et al., 2014a). In our study, lower MIC values (>6.25 μg/mL) of *Streptomyces* sp. YYS-7 were detected against the selected eight fungi expect for *C. acutatum* (ATCC 56815). Our previous study also showed that *Streptomyces* sp. SCA3-4 had high antifungal activities against 13 pathogenic fungi (Yang et al., 2012; Qi et al., 2019). It suggested that *Streptomyces* sp. can be a potential antifungal agent for inhibition of plant fungal diseases. The MIC difference of crude extracts from different *Streptomyces* might be related to their inherent profiles of metabolism and the kind of selected phytopathogen. In addition, the isolation of crude extracts using different extractive solvents was also important for antagonistic activity. We found that crude extracts isolated with 100% of ethyl alcohol owned the highest antagonistic activity to Foc TR4 in comparison of other concentration gradients (Figure 3A), whereas the extracts from *Streptomyces* sp. SCA3-4 and *S. lavendulae* strain SCA5 isolated by ethyl acetate exhibited the highest antifungal activity (Kumar et al., 2014b; Qi et al., 2019).

To further identify antifungal metabolites, a powerful analytical tool of GC-MS was used for compound analysis of microbial metabolites. A total of eleven chemical compounds identified from *Streptomyces* sp. YYS-7 mainly comprised of phenolic compounds, hydrocarbons, esters and acids (Supplementary Table S6). Phenolic compounds are commonly known as potent antimicrobial agents as well as antioxidant agents due to the removal capability of free radicals (Yogeswari et al., 2012). Recently, the antimicrobial compounds in the GC-MS fractions also contained the highest levels of phenolic substance (Kumar and Jadeja, 2018). Two phenolic compounds of 2,2′-methylenebis [6-(1,1-dimethylethyl)-4-methyl- and 2,4-Di-tert-butylphenol detected in *Streptomyces* sp. YYS-7 were reported as antimicrobial agents. The ester compounds were also detected in crude extracts of the strain YYS-7, including hexadecanoic acid, methyl ester, methyl stearate, and dibutyl phthalate. Hydrocarbon compounds were confirmed to possess an antagonistic potential against a wide range of pathogens (Elavarasi et al., 2014; Natarajan and Dhas, 2014). Acid compounds such as hexadecanoic acid and tetradecanoic acid also displayed both antioxidant and anticancer activities (Himaman et al., 2016). Hexadecanoic acid methyl ester isolated from the *Hibiscus sabdariffa Linn* can cause autolysis of membranous structures, induce aortic dilation and inhibit phagocytic activity and nitric oxide production of cells. Additionally, the accumulated evidences showed that methyl stearate had a high antibacterial and antioxidant ability (Ajoku et al., 2015; Tuhami et al., 2019). Thus, we proposed that these compounds could be the key contribution to antimicrobial activity of *Streptomyces* sp. YYS-7. Especially, some unknown function metabolites will be a subject of future investigation.

In pot experiment, *Streptomyces* sp. YYS-7 showed a biocontrol role on the decrease of banana wilt disease (Figure 5). Similarly, *Streptomyces caeruleatus* strain ZL2 significantly reduced the root rot of tomato seedlings caused by phytopathogenic Fusarium species (Zamoum et al., 2015). These metabolites of *Streptomyces* sp. can be predicted to play an important role on biocontrol efficiency (Ayyadurai et al., 2006; Faheem et al., 2015; Goudjal et al., 2016; Lu et al., 2016; Duan et al., 2020). Moreover, the antagonistic *Streptomyces* species exhibited a positive effect on the soil microbial structure (Zamoum et al., 2015). Interestingly, the fermentation broth of *Streptomyces* sp. YYS-7 also significantly increased the chlorophyll contents, plant heights and stem diameters of banana plantlets in comparison with the control groups 14 days after treatment. Biocontrol activities of *Streptomyces* are often associated with promotion of plant growth (El-Tarabily et al., 2009; Verma et al., 2011; Goudjal et al., 2016). Similar findings were showed that *Streptomyces* sp. significantly improved the biocontrol of Fusarium root rot disease and growth promotion of seedlings (El-Tarabily et al., 2009; Goudjal et al., 2016). Hence, the beneficial properties of *Streptomyces* sp. will provide a promising perspective for the possible exploration in the biocontrol field.

## Conclusion

In this study, the strain YYS-7 with a strong antifungal activity against Foc TR4 was isolated from the rhizosphere soil samples of plants in the primitive mountain. The morphological, cultural, physiological and biochemical characteristics were evaluated. Combining the alignment result of 16S *rRNA* sequence, the strain was assigned to *Streptomyces* sp. Treatment of with crude extracts of *Streptomyces* sp. YYS-7 resulted in Foc TR4 cell deformity and ultrastructure disappearing. Interestingly, the strain and its crude extracts also exhibited a broad-spectrum antifungal activity against other seven phytopathogens along with the lowest MIC (0.781 μg mL^–1^) against *C. fallax* (ATCC 34598) and the highest MIC (50 μg mL^–1^) against *C. acutatum Simmonds* (ATCC 56815). The GC-MS analysis revealed that eleven different compounds were identified from *Streptomyces* sp. YYS-7. Especially, crude extracts significantly improved the banana plants’ resistance to Foc TR4 in the pot experiment.

## Data Availability Statement

The datasets presented in this study can be found in online repositories. The names of the repository/repositories and accession number(s) can be found at: https://www.ncbi.nlm.nih.gov/nuccore/MN397826.

## Author Contributions

YW, JX, and WW developed the ideas and designed the experimental plans. DZ, JX, and WW supervised the research and provided the fund support. YW, YZ, DQ, KL, WT, and YC, TJ, and XZ were performed experiments. YW, YZ, TJ, XZ, and WW analyzed the data. YW and WW prepared the manuscript. All authors contributed to the article and approved the submitted version.

## Conflict of Interest

The authors declare that the research was conducted in the absence of any commercial or financial relationships that could be construed as a potential conflict of interest.

## Footnotes

1 https://www.ncbi.nlm.nih.gov/

2 https://www.ezbiocloud.net/identify

3 http://rsb.info.nih.gov/ij/

## Supplementary Material

TABLE S1 Summary of six plants selected for the rhizosphere soils.

TABLE S2 Morphological characteristics of the strain YYS-7.

TABLE S3 Nutrition utilization characteristics of the strain YYS-7.

TABLE S4 Antibiotic sensitivity test of the strain YYS-7.

TABLE S5 The spore germination rate of Foc TR4 treated with different concentrations of crude extracts of *Streptomyces* sp. YYS-7.

TABLE S6 The main component of crude extracts of *streptomyces* sp. YYS-7.

FIGURE S1 Identification of the strain YYS-7 by the morphological characteristics and the sequence alignment. **(A)** The morphological characteristics of the strain YYS-7 were detected by SEM. **(B)** A phylogenetic tree was constructed based on the 16S *rRNA* sequences from the strain YYS-7 and other selected strains.

FIGURE S2 Ultrastructure profiles of Foc TR4 cells by TEM. **(A)** Ultrastructure of Foc TR4 cells treated with 10% of DMSO. **(B–F)** Ultrastructure of Foc TR4 cells treated with crude extracts (25 μg mL^–1^) of *Streptomyces* sp. YYS-7.

FIGURE S3 Chemical structures of the identified compounds of *Streptomyces* sp. YYS-7 by GC-MS.

## References

[B1] Aamir M. Rai K. K. Zehra M. K. Samal S. Yadav M. Upadhyay R. S. (2020). “Endophytic actinomycetes in bioactive compounds production and plant defense system,” in *Microbial Endophytes*, eds Kumar A. Singh V. K., (Sawston: Woodhead Publishing), 189–229. 10.1016/b978-0-12-818734-0.00009-7

[B2] Ajoku G. A. Okwute S. K. Okogun J. I. (2015). Isolation of hexadecanoic acid methyl ester and 1,1,2-ethanetricarboxylic acid- 1-hydroxy-1, 1-dimethyl ester from the calyx of green Hibiscus Sabdariffa (Linn). *Nat. Prod. Chem. Res.* 3 1–5. 10.4172/2329-6836.1000169

[B3] Atta H. M. (2015). Biochemical studies on antibiotic production from *Streptomyces* sp.: taxonomy, fermentation, isolation and biological properties. *J. Saudi. Chem. Soc.* 19 12–22. 10.1016/j.jscs.2011.12.011

[B4] Ayyadurai N. Ravindra N. P. Sreehari R. M. Sunish K. R. Samrat S. K. Manohar M. (2006). Isolation and characterization of a novel banana rhizosphere bacterium as fungal antagonist and microbial adjuvant in micropropagation of banana. *J. Appl. Microbiol.* 100 926–937. 10.1111/j.1365-2672.2006.02863.x 16629993

[B5] Bakht J. Shaheen S. Shafi M. (2014). Antimicrobial potentials of Mentha longifolia by disc diffusion method. *Pak. J. Pharm. Sci.* 27 939–945. 25015464

[B6] Borrero C. Ordova’s J. Trillas M. Avilés M. (2006). Tomato Fusarium wilt suppressiveness. The relationship between the organic plant growth media and their microbial communities as characterised by Biolog. *Soil Biol. Biochem.* 38 1631–1637. 10.1016/j.soilbio.2005.11.017

[B7] Brewster D. T. Spiers A. G. Hopcroft D. H. (1997). Biocontrol of Phytophthora cactorumin in vitro with *Enterobacter aerogenes*. *New Zeal. J. Crop. Hort. Sci.* 25 9–18. 10.1080/01140671.1997.9513982

[B8] Chen Y. F. Chen W. Huang X. Hu X. Zhao J. T. Gong Q. (2013). Fusarium wilt-resistant lines of Brazil banana (*Musa* spp., AAA) obtained by EMS-induced mutation in a microcrosssection cultural system. *Plant Pathol.* 62 112–119. 10.1111/j.1365-3059.2012.02620.x

[B9] De Toledo L. G. Ramos M. A. Sposito L. Castilho E. M. Pavan F. R. Lopes E. O. (2016). Essential oil of *Cymbopogon nardus* (L.) rendle: a strategy to combat fungal infections caused by candida species. *Int. J. Mol. Sci.* 17:1252. 10.3390/ijms17081252 27517903 PMC5000650

[B10] Dewi T. Agustiyani D. Antonius S. Jakubiec-Krzesniak K. Rajnisz-Mateusiak A. Guspiel A. (2018). Secondary metabolites of actinomycetes and their antibacterial, antifungal and antiviral properties. *Pol. J. Microbiol.* 67 259–272. 10.21307/pjm-2018-048 30451442 PMC7256786

[B11] Duan G. Christian N. Schwachtje J. Walther D. Ebenhoh O. (2013). The metabolic interplay between plants and phytopathogens. *Metabolites* 3 1–23. 10.3390/metabo3010001 24957887 PMC3901261

[B12] Duan Y. Chen J. He W. (2020). Fermentation optimization and disease suppression ability of a Streptomyces ma. *FS-*4 from banana rhizosphere soil. *BMC Microbiol.* 20:24. 10.1186/s12866-019-1688-z PMC6995205 32005152

[B13] Elavarasi A. Peninal S. Rathna G. S. Kalaiselvam M. (2014). Studies on antimicrobial compounds isolated from mangrove endophytic fungi. *World J. Pharma. Pharm. Sci.* 3 734–744.

[B14] El-Tarabily K. A. Nassar A. H. Hardy G. E. S. J. Sivasithamparam K. (2009). Plant growth promotion and biological control of *Pythium aphanidermatum* a pathogen of cucumber, by endophytic actinomycetes. *J. Appl. Microbiol.* 106 13–26. 10.1111/j.1365-2672.2008.03926.x 19120624

[B15] Emmert E. Handelsman J. (1999). Biocontrol of plant disease: a (Gram-) positive perspective. *FEMS Microbiol. Lett.* 172 1–9. 10.1111/j.1574-6968.1999.tb13405.x 9987836

[B16] Faheem M. Raza W. Zhong W. Nan Z. Shen Q. Xu Y. (2015). Evaluation of the biocontrol potential of *Streptomyces goshikiensis* YCXU against *Fusarium oxysporum* f. sp. niveum. *Biol. Control* 81 101–110. 10.1016/j.biocontrol.2014.11.012

[B17] Fan Y. T. Chung K. R. Huang J. W. (2019). Fungichromin production by *Streptomyces padanus* PMS-702 for controlling cucumber Downy Mildew. *Plant Pathol. J.* 35 341–350. 31481857 10.5423/PPJ.OA.03.2019.0057 PMC6706012

[B18] Goudjal Y. Zamoum M. Sabaou N. Mathieu F. Zitouni A. (2016). Potential of endophytic *Streptomyces* spp. for biocontrol of *Fusarium* root rot disease and growth promotion of tomato seedlings. *Biocontrol Sci. Techn.* 26 1691–1075. 10.1080/09583157.2016.1234584

[B19] Guo G. Wang B. Z. Ma W. H. Li X. F. Yang X. L. Zhu C. H. (2013). Biocontrol of *Fusarium* wilt of banana: key influence factors and strategies. *Afr. J. Microbiol. Res.* 7 4835–4843. 10.5897/AJMR2012.2392

[B20] Hayakawa M. Nonomura H. (1987). Humic acid-vitamin agar, a new medium for the selective isolation of soil actinomycetes. *J. Ferment. Technolog.* 65 501–509. 10.1016/0385-6380(87)90108-7

[B21] Himaman W. Thamchaipenet A. Pathom-Aree W. Duangmal K. (2016). Actinomycetes from *Eucalyptus* and their biological activities for controlling *Eucalyptus leaf* and shoot blight. *Microbiol. Res.* 188-189 42–52. 10.1016/j.micres.2016.04.011 27296961

[B22] Kumar P. S. Al-Dhabi N. A. Duraipandiyan V. Balachandran C. Kumar P. P. Ignacimuthu S. (2014a). In vitro antimicrobial, antioxidant and cytotoxic properties of *Streptomyces lavendulae* strain SCA5. *BMC Microbiol.* 14:291. 10.1186/s12866-014-0291-6 25433533 PMC4265329

[B23] Kumar P. S. Duraipandiyan V. Ignacimuthu S. (2014b). Isolation, screening and partial purification of antimicrobial antibiotics from soil *Streptomyces* sp. SCA7. *Kaohsiung J. Med. Sci.* 30 435–446. 10.1016/j.kjms.2014.05.006 25224766 PMC11916805

[B24] Kumar R. R. Jadeja V. J. (2018). Characterization and partial purification of an antibacterial agent from halophilic actinomycetes *Kocuria* sp. strain rsk4. *Bioimpacts* 8 253–261. 10.15171/bi.2018.28 30397580 PMC6209832

[B25] Kumar S. Stecher G. Tamura K. (2016). Mega 7: molecular evolutionary genetics analysis version 7.0 for bigger datasets. *Mol. Biol. Evol.* 33 1870–1874. 10.1093/molbev/msw054 27004904 PMC8210823

[B26] Lam K. S. (2006). Discovery of novel metabolites from marine actinomycetes. *Curr. Opin. Microb.* 9 245–251. 10.1016/j.mib.2006.03.004 16675289

[B27] Leon J. Cuadra D. J. A. Galindo N. Jaramillo L. Vallejo M. Marguet E. (2016). Extracellular enzymes production and pathogen inhibitory activity of actinomycetes isolated from *Argopecten purpuratus*. *Rev. Biol. Mar. Oceanog.* 51 69–80. 10.4067/S0718-19572016000100007 27315006

[B28] Li Q. Chen X. Jiang Y. Jiang C. (2016). “Cultural, physiological, and biochemical identification of Actinobacteria,” in *Actinobacteria-Basics and Biotechnological Applications*, eds Dhanasekaran D. Jiang Y., (Norderstedt: BoD), 88–111.

[B29] Lou M. M. Zhu B. Muhammad I. Li B. Xie G. L. Wang Y. L. (2011). Antibacterial activity and mechanism of action of chitosan solutions against apricot fruit rot pathogen *Burkholderia seminalis*. *Carbohydr. Res.* 346 1294–1301. 10.1016/j.carres.2011.04.042 21605851

[B30] Lu D. Ma Z. Xu X. Yu X. (2016). Isolation and identification of biocontrol agent *Streptomyces rimosus* M527 against *Fusarium oxysporum* f. sp. cucumerinum. *J. Basic Microb.* 56 929–933. 10.1002/jobm.201500666 27192632

[B31] Mohseni M. Norouzi H. Hamedi J. (2013). Screening of antibacterial producing actinomycetes from sediments of the caspian sea. *Int. J. Mol. Med.* 2 64–71. PMC3920526 24551793

[B32] Natarajan V. Dhas A. (2014). Phytochemical composition and in vitro antimicrobial, antioxidant activities of ethanolic extract of *Leptadenia reticulata* [W&A] leaves. *Middle East J. Sci. Res.* 21 1698–1705. 10.5829/idosi.mejsr.2014.21.10.8596

[B33] Nguyen X. H. Naing K. W. Lee Y. S. Tindwa H. Lee G. H. Jeong B. K. (2012). Biocontrol potential of *Streptomyces griseus* H7602 against root rot disease (*Phytophthora capsici*) in pepper. *Plant Pathol. J.* 28 282–289. 10.5423/ppj.oa.03.2012.0040

[B34] Pridham T. G. Lyons A. J. (1961). *Streptomyces albus* (Rossi doria) Waksma et Henrici: taxonomic study of strains labelled *Streptomyces albus*. *J. Bacteriol.* 81 431–441. 10.1111/j.1365-2672.1961.tb00269.x 13737991 PMC279026

[B35] Qi D. F. Zou L. P. Zhou D. B. Chen Y. F. Gao Z. F. Feng R. J. (2019). Taxonomy and broad-spectrum antifungal activity of *Streptomyces* sp. SCA3-4 isolated from rhizosphere soil of *Opuntia stricta*. *Front. Microbiol.* 10:1390. 10.3389/fmicb.2019.01390 31316480 PMC6609889

[B36] Raza W. Ling N. Yang L. Huang Q. Shen Q. (2016). Response of tomato wilt pathogen *Ralstonia solanacearum* to the volatile organic compounds produced by a biocontrol strain *Bacillus amyloliquefaciens* SQR-9. *Sci. Rep.* 6:24856. 10.1038/srep24856 27103342 PMC4840334

[B37] Ruan J. S. Al-Tai A. M. Zhou Z. H. Qu L. H. (1994). *Actinopolyspora iraquiensis* sp. nov., A new halophilic actinomycete isolated from soil. *Int. J. syst. Bacteriol.* 44 759–763. 10.1099/00207713-44-4-759

[B38] Ruiz M. D. P. Ordóñez R. M. Isla M. I Esayago J. (2016). Activity and mode of action of *Parastrephia lepidophylla* ethanolic extracts on phytopathogenic fungus strains of lemon fruit from Argentine Northwest. *Postharvest Biol. Tech.* 114 62–68. 10.1016/j.postharvbio.2015.12.003

[B39] Sadeghian M. Bonjar G. H. S. Sirchi G. R. S. (2016). Postharvest biological control of apple bitter rot by soil-borne actinomycetes and molecular identification of the active antagonist. *Postharvest Biol. Tech.* 112 46–54. 10.1016/j.postharvbio.2015.09.035

[B40] Saitou N. Nei M. (1987). The neighbor-joining method: a new method for reconstructing phylogenetic trees. *Mol. Biol. Evol.* 4 406–425. 10.1093/oxfordjournals.molbev.a040454 3447015

[B41] Sajid I. Yao C. B. F. F. Shaaban K. A. Hasnain S. Laatsch H. (2009). Antifungal and antibacterial activities of indigenous *Streptomyces* isolates from saline farmlands: prescreening, ribotyping and metabolic diversity. *World J. Microb. Biot.* 25 601–610. 10.1007/s11274-008-9928-7

[B42] Sasaki T. Igarashi Y. Ogawa M. Furuma T. (2002). Identification of 6-prenylindole as an Antifungal metabolite of *Streptomyces* sp.TP-A0595 and synthesis and bioactivity of 6-substituted indoles. *J. Antibiot.* 5 1009–1012. 10.1002/chin.200319203 12546422

[B43] Shen Z. Ruan Y. Wang B. Zhong S. Su L. Li R. (2015). Effect of biofertilizer for suppressing *Fusarium* wilt disease of banana as well as enhancing microbial and chemical properties of soil under greenhouse trial. *Appl. Soil Ecol.* 93 111–119. 10.1016/j.apsoil.2015.04.013

[B44] Shirling E. B. Gottlieb D. (1966). Methods for characterization of *Streptomyces* species. *Int. J. Syst. Evol. Microbiol.* 16 313–340. 10.1099/00207713-16-3-313

[B45] Shivlata L. Satyanarayana T. (2015). Thermophilic and alkaliphilic Actinobacteria: biology and potential applications. *Front. Microbiol.* 6:1014. 10.3389/fmicb.2015.01014 26441937 PMC4585250

[B46] Shomura T. Yoshida J. Amano S. Kojima M. Inouye S. Niida T. (1979). Studies on Actinomycetales producing antibiotics only on agar culture. I. Screening, taxonomy and morphology-productivity relationship of *Streptomyces halstedii*, strain SF-1993. *J. Antibiot.* 32 427–435. 10.7164/antibiotics.32.427 528390

[B47] Singh D. P. Patil H. J. Prabha R. Yandigeri M. S. Prasad S. R. (2018). Actinomycetes as potential plant growth-promoting microbial communities. *Crop Impro. Microb. Biotech.* 2018 27–38. 10.1016/B978-0-444-63987-5.00002-5

[B48] Singh S. P. Gaur R. (2016). Evaluation of antagonistic and plant growth promoting activities of chitinolytic endophytic actinomycetes associated with medicinal plants against Sclerotium rolfsii in chickpea. *J. Appl. Microb.* 121 506–518. 10.1111/jam.13176 27170067

[B49] Soltanzadeh M. Soltani Nejad M. Shahidi Bonjar G. H. (2016). Application of soil-borne actinomycetes for biological control against fusarium wilt of chickpea (*Cicer arietinum*) caused by *Fusarium solani* f. sp. pisi. *J. Phytopathol.* 164 967–978. 10.1111/jph.12517

[B50] Song S. Chen X. Huang D. Xu Y. Zeng H. Hu X. (2016). Identification of miRNAs differentially expressed in fusarium wilt-resistant and susceptible banana varieties. *S. Afr. J. Bot.* 106 244–249. 10.1016/j.sajb.2016.06.007

[B51] Supaphon P. Phongpaichit S. Rukachaisirikul V. Sakayaroj J. (2013). Antimicrobial potential of endophytic fungi derived from three seagrass species: *Cymodocea serrulata*, *Halophila ovalis* and *Thalassia hemprichii*. *PLoS One* 8:e0072520. 10.1371/journal.pone.0072520 23977310 PMC3745589

[B52] Tuhami E. H. Adam I. A. Almain A. Mohammed M. M. (2019). Phytochemical screening, GC-MS analysis, antibacterial and antioxidant activity of seeds oil of *Annona squmosa* L. Sudanese medicinal plant. *J. Pharm. Pharmcol.* 7 1–6.

[B53] Valli S. Suvathi S. S. Aysha O. S. Nirmala P. Vinoth K. P. Reena A. (2012). Antimicrobial potential of *Actinomycetes* species isolated from marine environment. *Asian Pac. J. Trop. Biomed.* 2 469–473. 10.1016/S2221-1691(12)60078-1 23569952 PMC3609324

[B54] Verma V. C. Singh S. K. Prakash S. (2011). Bio-control and plant growth promotion potential of siderophore producing endophytic *Streptomyces* from *Azadirachta indica* A. *Juss. J. Basic Microb.* 51 550–556. 10.1002/jobm.201000155 21656792

[B55] Wang L. Y. Xing M. Y. Di R. Luo Y. P. (2015). Isolation, identification and antifungal activities of *Streptomyces aureoverticillatus* HN6. *J. Plant Pathol. Microb.* 6 1–5. 10.4172/2157-7471.1000281

[B56] Wang W. Hu Y. Sun D. Staehelin C. Xin D. Xie J. (2012). Identification and evaluation of two diagnostic markers linked to *Fusarium* wilt resistance (race 4) in banana (*Musa* spp.). *Mol. Biol. Rep.* 39 451–459. 10.1007/s11033-011-0758-6 21547366

[B57] Wang X. N. Radwan M. M. Taráwneh A. H. Gao J. T. Wedge D. E. Rosa L. H. (2013). Antifungal activity against plant pathogens of metabolites from the endophytic fungus *Cladosporium cladosporioides*. *J. Agric. Food Chem.* 61 4551–4555. 10.1021/jf400212y 23651409 PMC3663488

[B58] Wang Y. Xia Q. Wang G. Zhang H. Lu X. Sun J. (2017). Differential gene expression in banana roots in response to *Fusarium* wilt. *Can. J. Plant Pathol.* 39 163–175. 10.1080/07060661.2017.1342693

[B59] Williams S. T. Goodfellow M. Alderson G. Wellington E. M. H. Sneath P. H. A. Sackin M. J. (1983). Numerical classification of *Streptomyces* and related genera. *J. Gen. Microbiol.* 129 1743–1813. 10.1099/00221287-129-6-1743 6631406

[B60] Xu J. Zhao X. Han X. Du Y. (2007). Antifungal activity of oligochitosan against Phytophthora capsici and other plant pathogenic fungi *in vitro*. *Pestic. Biochem. Phys.* 87 220–228. 10.1016/j.pestbp.2006.07.013

[B61] Yang D. Liu H. Y. Gao W. W. Chen S. L. (2010). Activities of essential oils from *Asarum heterotropoides* var. mandshuricum against five phytopathogens. *Crop Prot.* 29 295–299. 10.1016/j.cropro.2009.12.007

[B62] Yang X. J. Fang M. Yao Y. Cao F. J. Rui Y. Ma Y. N. (2012). In vitro antifungal activity of sanguinarine and chelerythrine derivatives against phytopathogenic fungi. *Molecules* 17 13026–13035. 10.3390/molecules171113026 23124471 PMC6268840

[B63] Yogeswari S. Ramalakshmi S. Neelavathy R. Johnpaul M. (2012). Identification and comparative studies of different volatile fractions from *Monochaetia kansensis* by GC-MS. *Glob. J. Pharmacol.* 6 65–71.

[B64] Yoon S. H. Ha S. M. Kwon S. Lim J. Chun J. (2017). Introducing EzBioCloud: a taxonomically united database of 16S rRNA gene sequences and whole-genome assemblies. *Int. J. Syst. Evol. Micr.* 67 1613–1617. 10.1099/ijsem.0.001755 28005526 PMC5563544

[B65] Yun T. Y. Feng R. J. Zhou D. B. Pan Y. Y. Chen Y. F. Wang F. (2018). Optimization of fermentation conditions through response surface methodology for enhanced antibacterial metabolite production by *Streptomyces* sp. 1-14 from cassava rhizosphere. *PLoS One* 13:e0206497. 10.1371/journal.pone.0206497 30427885 PMC6241123

[B66] Zamoum M. Goudjal Y. Sabaou N. Barakate M. Mathieu F. Zitouni A. (2015). Biocontrol capacities and plant growth-promoting traits of endophytic actinobacteria isolated from native plants of Algerian Sahara. *J. Plant Dis. Protect.* 122 215–223. 10.1007/BF03356555

[B67] Zapata-Sarmientoa D. H. Palacios-Palaa E. F. Rodríguez-Hernándezb A. A. Melchora D. L. M. Rodríguez-Monroya M. Sepúlveda-Jiménez G. (2019). *Trichoderma asperellum*, a potential biological control agent of *Stemphylium vesicarium*, on onion (*Allium cepa* L.). *Biol. Control* 140 1–9. 10.1016/j.biocontrol.2019.104105

