# Supplementary material for: A Newly Isolated Streptomyces sp. YYS-7 With a Broad-Spectrum Antifungal Activity Improves the Banana Plant Resistance to Fusarium oxysporum f. sp. cubense Tropical Race 4
Source: Front Microbiol. 2020 Aug 12;11:1712. doi: 10.3389/fmicb.2020.01712 (PMC7438861; doi:10.3389/fmicb.2020.01712)
Supplement: TABLE S1 — Summary of six plants selected for the rhizosphere soils. [file Table_1.DOCX]

**Table S1. Summary of six plants selected for the rhizosphere soils**

| **Plant name** | **Sampling location** |
| --- | --- |
| *Machilus pingii* | 109°43’05” E, 19°02’03” N |
| *Castanopsis hystrix* | 109°43’16” E, 19°02’00” N |
| *Syzygium cumini* | 109°43’18” E, 19°02’00” N |
| *Lithocarpus glaber* | 109°43’18” E, 19°02’00” N |
| *Cinnamomum porrectum* | 109°4340” E, 19°02’91” N |
| *Lithocarpus corneus* | 109°43’01” E, 19°02’57” N |

**Table S2. Morphological characteristics of the strain YYS-7**

| **Characteristics** | **Results** |
| --- | --- |
| Aerial mycelium color | White |
| Substrate mycelium color | Lemon Chiffon |
| Diffusible pigment | - |
| Melanin pigment | - |
| Spore chain morphology | Straight to rectiflexibiles |
| Spore surface | Rugose |

**Table S3. Nutrition utilization characteristics of the strain YYS-7**

| **Characteristics** | **Result** |
| --- | --- |
| **Carbon test items** |  |
| D-Fructose | + |
| D-Xylose | + |
| Rhamnose | + |
| arabinose | + |
| Raffinose | - |
| Melezitose | + |
| Galactose | + |
| α- Lactose | + |
| Sucrose | + |
| D- Mannose | + |
| Sorbitol | + |
| Inositol | + |
| D-Mannitol | + |
| maltose | + |
| Starch | + |
| Melibiose | + |
| **Nitrogen test items** |  |
| **Histidine** | + |
| **Methionine** | + |
| **Serine** | + |
| Glycine | + |
| **Hydroxyproline** | - |
| Phenylalanine | + |
| Glutamate | - |
| **Cysteine** | + |
| **Arginine** | + |
| **Valine** | + |
| Tyrosine | + |
| Ammonium acetate | - |
| Ammonium nitrate | - |
| Asparagine | + |
| L-Phenylalanate | + |
| Salicylic acid | - |
| Anhydrous creatine | + |

Note: +, Positive for test; −, Negative for test

**Table S4. Antibiotic sensitivity test of the strain YYS-7**

| Antibiotic sensitivity（μg disc^-1^） | **Results** |
| --- | --- |
| Clindamycin (2) | R |
| Chloramphenicol (30) | R |
| Furazolidone (300) | S |
| Compound Sulfamethoxazole (1.25) | S |
| Polymyxin B (300 IU/disc) | S |
| Vancomycin (30) | S |
| Ciprofloxacin (5) | S |
| Ofloxacin (50) | S |
| Norfloxacin (10) | R |
| Penicillin (10U/disc) | S |
| Erythromycin (15) | S |
| Minocycline (30) | S |
| Doxycycline (30) | R |
| Tetracycline (30) | S |
| Neomycin (30) | R |
| Kanamycin (30) | S |
| Gentamicin (10) | R |
| Amikacin (30) | S |
| Cefoperazone (75) | S |
| Ceftriaxone (30) | S |
| Ceftazidime (30) | R |
| Cefuroxime (30) | S |
| Cefradine (30) | R |
| Cefazolin (30) | R |
| Cephalexin (30) | S |
| Midecamycin (30) | R |
| Carbenicillin (100) | S |
| Ampicillin (10) | S |
| Oxacillin (1) | R |
| Piperacillin (100) | R |

Note: S for Sensitivity; R for Resistance.

**Table S5. The spore germination rate of Foc TR4 treated with different concentrations of crude extracts of *Streptomyces* sp. YYS-7**

| **Concentration (µg mL^-1^)** | | **Inhibition rate of spore germination (%)** |
| --- | --- | --- |
| 6.25 | 27.70 ± 4.03a | |
| 12.5 | 37.90 ± 9.19b | |
| 25 | 44.40 ± 7.21ab | |
| 50 | 53.20 ± 4.46c | |
| 100 | 59.20 ± 0.95dc | |
| 200 | 70.50 ± 2.46e | |
| 400 | 80.40 ± 4.06ef | |
| 600 | 91.50 ± 1.00g | |

**Table S6. The main component of crude extracts of *streptomyces* sp. YYS-7**

| **Compounds** | **RT (min)** | **MM** | **Area (%)** | **MF** | **Activity** |
| --- | --- | --- | --- | --- | --- |
| Hexadecanoic acid, methyl ester | 32.992 | 270 | 4.79% | C_17_H_34_O_2_ | Antioxidant |
| 2,4-Di-tert-butylphenol | 35.257 | 206 | 5.23% | C_14_H_22_O |  |
| Methyl stearate | 35.257 | 298.287 | 1.84% | C_19_H_38_O_2_ | Antimicrobial |
| Tetradecanoic acid | 42.002 | 228.209 | 8.16% | C_14_H_28_O_2_ | Antifungal |
| Dibutyl phthalate | 43.227 | 278.152 | 23.18% | C_16_H_22_O_2_ | No activity reported |
| Pentadecanoic acid | 43.998 | 242.225 | 6.40% | C_15_H_30_O_2_ | No activity reported |
| n-Hexadecanoic acid | 45.911 | 256.24 | 37.71% | C_16_H_32_O_2_ | Antibacterial |
| Cetene | 48.176 | 224.25 | 1.55% | C_16_H_32_ | No activity reported |
| Phenol,2,2'-methylenebis[6-(1,1-dimethylethyl)-4-methyl- | 51.263 | 340.24 | 9.72% | C_17_H_34_O_2_ | No activity reported |
| Cyclohexanebutanoic acid | 48.176 | 170.131 | 1.55% | C_14_H_22_O | No activity reported |
| Heptadecanoic acid | 47.798 | 224.25 | 2.17% | C_19_H_38_O_2_ | No activity reported |

RT, retention time; MM, molecular mass of compounds; MF, molecular formula.
