# Supplementary figures and images for: A Newly Isolated Streptomyces sp. YYS-7 With a Broad-Spectrum Antifungal Activity Improves the Banana Plant Resistance to Fusarium oxysporum f. sp. cubense Tropical Race 4
Source: Front Microbiol. 2020 Aug 12;11:1712. doi: 10.3389/fmicb.2020.01712 (PMC7438861; doi:10.3389/fmicb.2020.01712)

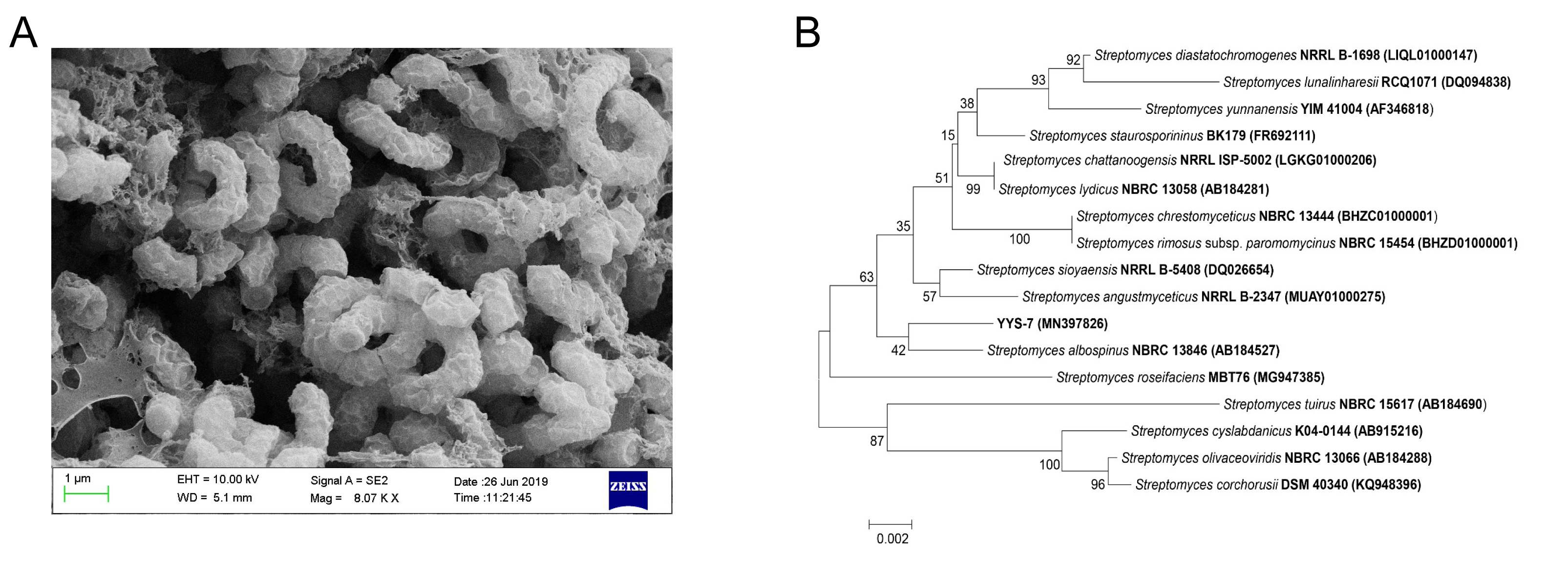

Supplement: FIGURE S1 — Identification of the strain YYS-7 by the morphological characteristics and the sequence alignment. (A) The morphological characteristics of the strain YYS-7 were detected by SEM. (B) A phylogenetic tree was constructed based on the 16S rRNA sequences from the strain YYS-7 and other selected strains. [file Image_1.TIF]

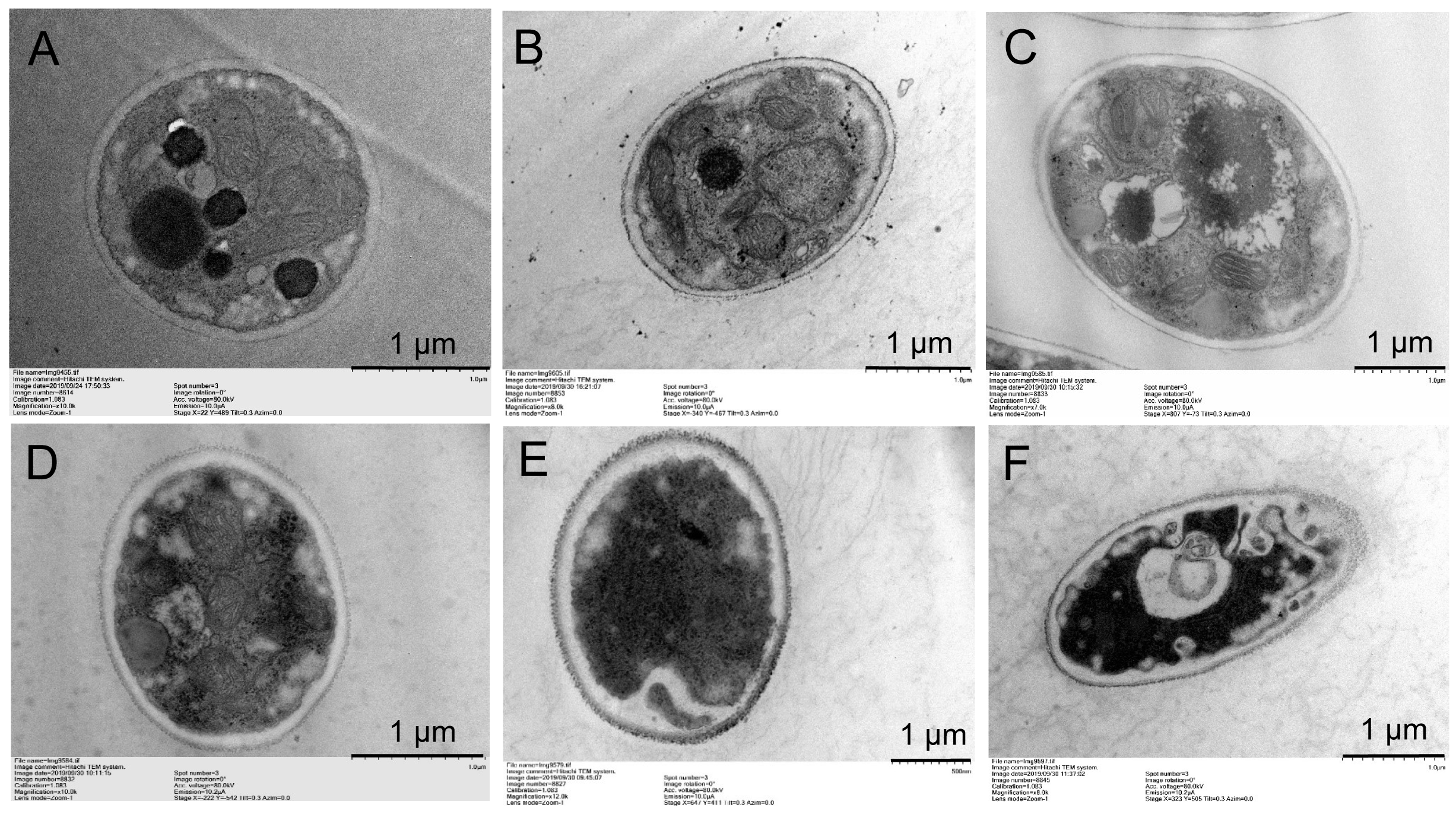

Supplement: FIGURE S2 — Ultrastructure profiles of Foc TR4 cells by TEM. (A) Ultrastructure of Foc TR4 cells treated with 10% of DMSO. (B–F) Ultrastructure of Foc TR4 cells treated with crude extracts (25 μg mL–1) of Streptomyces sp. YYS-7. [file Image_2.TIF]

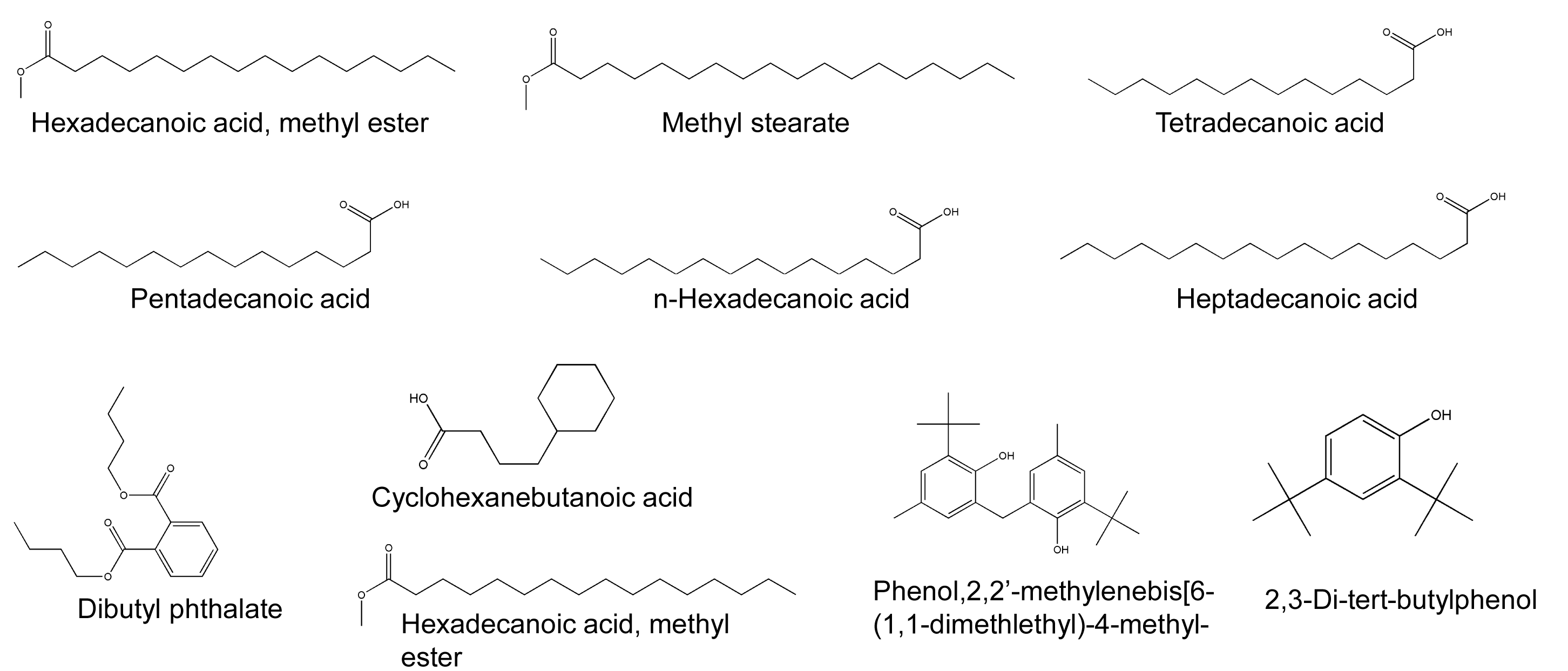

Supplement: FIGURE S3 — Chemical structures of the identified compounds of Streptomyces sp. YYS-7 by GC-MS. [file Image_3.TIF]
